# From Clinical Encounter to Draft Documentation: A Mechanistic Narrative Review of Ambient Scribe Technology

**DOI:** 10.7759/cureus.110201

**Published:** 2026-06-03

**Authors:** Thomas W Kuhn

**Affiliations:** 1 Behavioral Health Services, Holland Hospital, Holland, USA

**Keywords:** ambient scribe, artificial intelligence (ai) in medicine, automatic speech recognition, clinical documentation, clinician workflow, informed consent, large language models, medical informatics

## Abstract

Clinicians and health systems increasingly use ambient scribe tools to generate draft clinical notes in an effort to reduce documentation burden, enhance workflow, and improve clinician and patient experience. Safe and effective use is enhanced by understanding that the draft note is not a direct record of the encounter, but the product of a multistage representational process in which clinical sound is captured, converted into digital data, transformed into text, and reshaped into documentation. This narrative review examined literature from November 2025 through May 2026 using PubMed, IEEE Xplore, IsisCB Explore, ACM Digital Library, arXiv, Google Scholar, and citation tracking, prioritizing peer-reviewed sources with selective inclusion of foundational technical and conceptual works. The review translates a clinician-accessible account of the ambient scribe pipeline into practical guidance for clinician use. Privacy, consent, and medicolegal issues are also considered, including remote data transmission and clinician responsibility for the final note. This analysis leads to the 3C approach: Choose, Capture, Check. This framework emphasizes clinician decision-making before the encounter, deliberate communication and capture practices during the encounter, and targeted review after note generation.

## Introduction and background

Clinicians and healthcare systems are increasingly adopting ambient scribe tools to reduce documentation burden and address clinician burnout [1-4]. These tools capture sound generated during clinical encounters and process it into draft clinical notes. They differ from traditional speech-recognition dictation systems, in which the clinician actively dictates the note into a microphone. Ambient scribes instead process captured clinical conversation through multiple computational stages and generate a draft note for clinician review.

Emerging evidence suggests that ambient scribes may reduce documentation time and improve aspects of clinician experience and workflow [1,2,5-10]. A recent large, multisite study found modest reductions in total electronic health record (EHR) time and documentation time after adoption of ambient scribes, along with a slight increase in visit volume, with variation in benefit across specialties and clinician characteristics [10]. At the same time, evaluation studies have reported variable note quality, omission of clinically relevant information, speaker misattribution, and generation of inaccurate or unsupported text [1,11-16]. A recent study found that 70% of draft notes contained at least one error, and another found that, on average across platforms, 26.3% of evaluated key clinical elements were omitted or erroneous [11,16].

The shift from traditional dictation to ambient capture and note generation has practical consequences. Ambient scribe output may be affected by acoustic capture, speaker attribution, transcript formation, and note generation [11-13,16-18]. Ambient scribes also change the privacy, consent, and medicolegal context because documentation now depends in part on captured and processed sound generated during the clinical encounter, not solely on clinician-authored or clinician-dictated text [3,17,19,20]. In response to the potential benefits, limitations, and risks, prior authors and professional discussions have emphasized that clinicians should review, edit, and approve draft notes before incorporation into the medical record, and that health systems should accompany implementation with appropriate evaluation and governance [1,3,12,19].

These recommendations are appropriate but incomplete. A broad instruction to review the draft does not tell clinicians how the note was produced, why particular artifacts arise, or where the note is most vulnerable to loss or distortion of clinical meaning. Literature in cognitive science and human-computer interaction suggests that a functional mental model of how complex tools operate supports more effective use [21-23]. Applied to ambient scribes, that principle suggests that clinicians need a practical understanding of the pipeline by which captured sound becomes a transcript, a transcript becomes a draft note, and a draft note becomes a signed clinical document.

Although individual studies have described ambient scribe performance, implementation, note quality, and associated risks, the peer-reviewed literature has not yet provided a concise, clinician-oriented synthesis that explains how ambient scribes produce draft notes, links that process to characteristic artifacts, and translates those links into practical guidance for clinical use [1,2,4,12,24,25]. To address that gap, this review provides a clinician-accessible account of how ambient scribes work, examines major fidelity, privacy, consent, and medicolegal considerations, and derives guidance for clinical use. The review concludes with the 3C approach to ambient scribe use: Choose, Capture, Check. This approach translates the preceding analysis into three categories of clinician action: choosing whether the tool is appropriate before the encounter, optimizing capture of clinically important information during the encounter, and checking the generated draft after note generation for predictable artifacts.

An earlier draft of this article was previously posted to the JMIR preprint server on May 8, 2026 [26].

## Review

Methods

Review Design and Search Strategy

This review used a narrative review methodology because the purpose was to develop an integrated, clinician-accessible explanation of a complex technology, rather than to produce a pooled estimate of effect [27-29]. The review drew on technical, clinical, human factors, patient safety, privacy, consent, and medicolegal literature.

The author conducted literature searches between November 2025 and May 2026 in PubMed, IEEE Xplore, IsisCB Explore, ACM Digital Library, arXiv, and Google Scholar. Additional sources were identified through backward and forward citation tracking. Searches were iterative and concept-driven rather than governed by a prespecified systematic review protocol. Early searching identified a recurring pipeline structure: clinical sound is captured, converted into digital signal data, processed into text through automatic speech recognition and related methods, transformed into draft documentation, and then reviewed by the clinician. This pipeline model guided focused searches on the stages of ambient scribe systems, their underlying technology, empirically described documentation artifacts, clinician interaction with draft notes, and downstream privacy, consent, and medicolegal implications.

Source Selection, Conceptual Mapping, and Scope

Sources were included when they clarified one or more elements of the ambient scribe pipeline, documented observed note-quality limitations, supported plausible mechanisms of error, or explained implications for clinical use. Peer-reviewed sources were prioritized. Foundational technical works, textbooks, standards documents, historical sources, and selected preprints were included when they provided necessary conceptual or technical context not otherwise available in the peer-reviewed literature. Formal risk-of-bias assessment and quantitative pooling were not performed because the goal was explanatory synthesis rather than exhaustive evidence enumeration.

Analysis proceeded through iterative mapping of the literature onto the developing pipeline framework. Sources were used to identify major pipeline stages, recurring categories of documentation artifact, and practical implications for clinicians using ambient scribe output. Practical recommendations were then organized into the 3C approach to ambient scribe use: Choose, Capture, Check.

Analytic Perspective and Conceptual Sufficiency

The review was informed by the author’s longitudinal clinical experience with handwritten notes, typed notes, human transcription, speech-recognition dictation, and ambient scribe systems, as well as earlier technical study in engineering, programming, and digital logic. This background shaped the review’s emphasis on mechanisms, documentation artifacts, and clinician response. Technical sources needed to understand the ambient scribe pipeline were synthesized in the manuscript selectively, at a level useful for clinical interpretation and practical use.

The search was considered conceptually sufficient when additional searching no longer materially changed the understanding of the major pipeline stages, recurring artifact categories, or practical implications for clinician use. 

The ambient scribe pipeline

Overview

Commercially available ambient scribe systems begin with sound generated during the clinical encounter. That sound is converted into digital data, some or all of the resulting data are transmitted for processing, and draft documentation is returned to the clinician for review. In this context, “digital” means that information is represented by combinations of the digits 0 and 1, in a form that computers can store, transmit, and process. Once sound from the encounter has been converted into digital data, each later stage works from digital data produced by the prior stage, not from direct access to the encounter itself.

**Figure 1:**
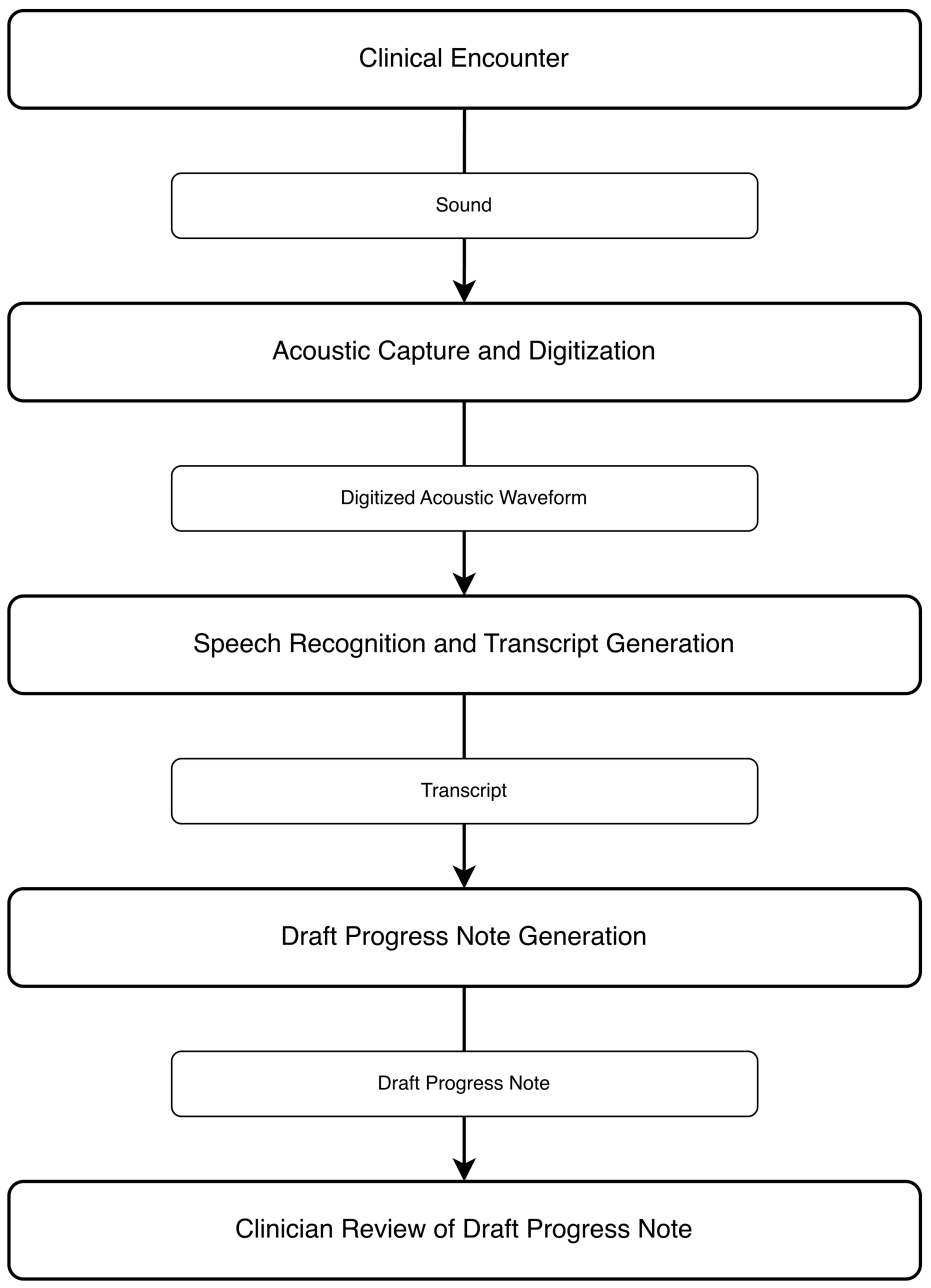
Schematic representation of the core computational pipeline and associated clinician workflow in ambient scribe use Large rectangles indicate major workflow stages, and small rectangles indicate outputs passed between stages.

Although commercial ambient scribe products vary in implementation, their general operation can be understood as a common functional pipeline [18,30,31]. The major stages are acoustic capture and digitization; speech-to-text processing and transcript generation; and draft progress note generation [12,18,31,32]. The output of each stage becomes the input to the next. As a result, information that is missed, distorted, or misassigned early in the process may be carried forward into later stages [12,16]. The pipeline and associated clinician workflow are represented schematically in Figure 1, and described more fully below.

Acoustic Capture and Digitization

In the first stage, microphones embedded in devices such as smartphones, tablets, or laptops capture sound generated during the clinical encounter [18,24,33]. This includes the voices of the clinician, patient, and other participants, room noise, and other incidental sounds. The microphones convert this sound into a continuously varying electrical signal. The device then digitizes that signal by measuring its amplitude thousands of times per second and representing those measurements as numerical values [33-36]. The output of this stage is a digital representation of the sound captured by the microphone. In some products, this output is stored as a recording before being sent to the next stage; in others, the representation is processed in real time. In either case, later stages depend on what was verbalized, acoustically captured, and digitized. Clear verbalization of important clinical material, adequate microphone placement, and attention to the acoustic environment provide higher-quality input for later processing stages.

Speech-to-Text Processing and Transcript Generation

In the second stage, the digitized acoustic representation is converted into a written transcript via automatic speech recognition. This technology involves computational analysis of patterns in the digital signal that correspond probabilistically to spoken language [36-42]. More than one written sequence may be plausible for a given portion of audio, especially when speech is rapid, interrupted, overlapping, accented, partially obscured by noise, or clinically specialized. The resulting transcript reflects a text sequence supported by both the acoustic signal and statistical associations derived from prior data [36,38-42].

Additional processing may classify portions of the signal as speech, silence, or nonspeech sound; group stretches of speech likely produced by the same participant; and associate those participants with clinically meaningful roles such as clinician, patient, or other speaker [12,13,43,44]. If those associations are inaccurate or incomplete, the words may be preserved while their source is misassigned. For example, a clinician’s question may be carried forward as though it were the patient’s statement. The resulting transcript of the encounter becomes the main textual input for draft progress note generation. 

Draft Progress Note Generation and Transmission to Clinician

In the third stage, the transcript is transformed into draft clinical documentation. The transcript is first divided into smaller text units and converted into numerical representations that can be handled mathematically [45-48]. Those representations allow relationships among words, phrases, surrounding context, and statistical patterns derived from large bodies of text to be calculated.

Draft text is then produced through probabilistic language modeling. At each step, likely continuations of the existing sequence are predicted, text is appended, and the sequence is extended until a stopping condition is reached [49-51]. In ambient scribe systems, this generation is conditioned not only on the encounter transcript, but also on instructions about the desired output, such as whether the note should follow a particular documentation format [31,49]. The resulting output is transmitted to the clinician as a draft progress note for review. Although the output may be described in practice as a summary of the encounter, it is better understood as a draft clinical note shaped by the transcript, formatting instructions, and statistical patterns derived from large datasets.

Because language models are optimized to generate plausible, coherent text, the resulting note may read smoothly even when its clinical content requires correction. A well-written draft may reduce documentation burden, but it still requires targeted clinician review for fidelity to the clinical encounter.

Pipeline artifacts and downstream effects

Because each stage operates on the output of the stage before it, documentation artifacts may arise during capture, transcription, speaker attribution, or note generation, and may compound as they are processed through the pipeline [12,17,18]. A clinician who understands this is better positioned to use ambient scribes effectively. Specific vulnerabilities are discussed below.

Capture and Transcription Artifacts

The first vulnerability is input formation: what becomes usable input to the system, and how accurately the spoken content is transcribed once it does. Ambient scribes work best when clinically important information is verbalized clearly and captured with adequate acoustic fidelity.

Clinically relevant information may be absent or underrepresented in the computational input if it is observed but not verbalized, conveyed through gesture, mentioned only in side conversation, expressed ambiguously, embedded in overlapping speech, or left as tacit clinical reasoning rather than spoken assessment. Background noise, interruptions, variable microphone position, overlapping voices, and conversational speech may also degrade transcription quality [14,18,32,52].

These risks are not evenly distributed across patient populations. Recent automatic speech recognition work found poorer transcription performance for Black patients than for White patients in home-health encounters [53]. Although this work did not evaluate full ambient scribe deployments, it supports the broader point that the transcription process itself can be a source of inequity and error, rather than a neutral preprocessing step [17]. Similar concerns may apply to encounters involving limited English proficiency, interpreters, dysarthria, atypical speech patterns, or languages and accents underrepresented in training data, although the ambient-scribe literature has not yet quantified all of these risks. Clinicians should therefore consider capture quality not only in terms of room acoustics, but also in terms of which patient populations and encounter types may face elevated risk of transcription error [17,32,53].

Omission deserves particular attention during review. In a simulated evaluation of five ambient platforms, omission errors were the most common note defect, accounting for roughly three-quarters of all observed errors [11]. This is important because omissions may be harder to detect than obviously incorrect insertions. A draft that omits a pertinent negative, qualifying detail, physical examination finding, or element of clinical reasoning may not call attention to what is missing.

Speaker Attribution Artifacts

A second vulnerability is speaker attribution. A clinically useful note requires not only the correct words, but also the correct identification of who said them. Systems that segment speech, group speech by speaker, or assign roles such as clinician, patient, or family member may preserve the wording of a statement while misassigning its source [12,43,44]. In clinical documentation, this may convert a clinician’s question into a patient-reported fact, or transform family history into the patient’s personal history. Attribution errors may be difficult to detect because the note may read naturally even when the clinical meaning has been materially changed.

Note Generation Artifacts

After a transcript is produced, the note-generation stage can introduce additional artifacts. Information from the transcript is filtered, condensed, weighted, and arranged into the form of a clinical note. At this stage, artifacts may include unsupported or incorrect content, including reversal of clinical meaning when a denied symptom or absent finding is stated or implied to be present; overstated certainty; overconfident completion of familiar clinical patterns; and inappropriate prioritization or disproportionate emphasis of secondary concerns [11,13-17,54,55]. The fluency of the output creates a challenge for clinician review: a note that reads as if a clinician wrote it may discourage the scrutiny needed to detect these errors.

Editing Burden

A clinically useful draft must not only be factually accurate; it must also fit the clinician’s documentation style and review process. A draft that is too long, poorly organized, stylistically mismatched, or difficult to edit may reduce the practical benefit of the tool even when its content is correct.

In a usability study, unedited draft notes generated by ambient scribes scored worse than manual summaries on the 9-Item Physician Documentation Quality Instrument (PDQI), had higher word counts, and had lower lexical diversity; users also criticized their greater length and rigid structure [56]. Another study found that physicians were often positive about workload and patient engagement but negative about note length, editing requirements, and stylistic fit [57]. A qualitative study examining implementation across a large academic medical center found that note formatting and loss of personal documentation voice were recurring concerns, with clinicians reporting that ambient scribe output did not adequately reflect their clinical reasoning or communication style [58].

Ambient scribes may save time for some users, but for others, they may shift work downstream by producing drafts that require substantial revision [5,8,9,57]. The largest multisite study to date found modest average time savings that varied meaningfully by specialty and clinician characteristics, with primary care clinicians benefiting more than specialists, and no reduction in after-hours EHR time [10]. Efficiency depends not only on whether the system generates a draft, but on whether that draft is accurate, appropriately structured, and easy enough for the clinician to review and edit.

Taken together, these observations show why efficient review of ambient scribe output is facilitated by an approach that targets predictable artifacts of the pipeline [11,13,15-17].

Privacy, Consent, and Medicolegal Considerations

Because ambient scribes depend on capture and processing of encounter-derived data, responsible implementation requires attention to disclosure, consent, data handling, retention, and clinician review. These issues extend beyond the generated note itself. Encounter audio may be recorded, and downstream data may be transmitted, transformed, and potentially retained and used beyond the immediate documentation task [17,19].

Patients may reasonably want to know whether audio is recorded, whether recordings or transcripts are retained, who can access recordings, transcripts, draft notes, or other derived materials, whether those materials are used for system improvement or model training, and whether they may decline use without compromising care [17,19,59-61]. Privacy concerns may also extend to individuals other than the patient. Clinical conversations often include references to family members, caregivers, household contacts, and others who may not be present and/or have not consented to recording, processing, or documentation of information about them. A recent study found that generated notes may include third-party personal information from transcripts, introducing a privacy risk not fully addressed by patient-centered consent alone [62].

Although statutory requirements for recorded conversations vary across US jurisdictions, ethical and professional guidance supports meaningful patient consent before ambient scribe use [19,59-61,63]. Consent is enabled when patients understand that the encounter may be recorded or otherwise captured, that information from the encounter will be processed into a draft note, that the draft may require clinician correction, and that data handling and retention practices may vary [19,59-61].

Clinicians remain responsible for the content of notes they sign, including notes generated with ambient scribe assistance [17,19,60]. Errors in draft documentation may affect downstream clinical decision-making, billing and coding, and liability exposure [17,19]. Medicolegal implications may extend beyond the signed note itself. Acoustic recordings, transcripts, draft notes, metadata, and other processing artifacts retained by vendors or health systems may persist outside the clinical record and become relevant in litigation or regulatory review [17,19]. These considerations support deliberate implementation and informed clinician use.

The 3C approach to ambient scribe use: Choose, Capture, Check

Ambient scribes are best used selectively, with attention to patient, encounter, documentation, and workflow fit. A mechanistic understanding of the pipeline supports an encounter-level approach: Choose, the pre-capture decision about whether ambient scribe use is appropriate; Capture, the structuring of the encounter so clinically important information enters the pipeline in usable form; and Check, targeted review of the generated draft with attention to predictable artifacts of the pipeline. These steps address different parts of the pipeline. Clear verbalization, identification of speakers when needed, and attention to microphone placement can improve capture and reduce ambiguity, but some artifacts may still appear despite careful encounter technique. These include transcription uncertainty, speaker-attribution error, unsupported or incorrect content, misplaced emphasis, and preservation of an earlier statement after later correction. For that reason, the framework emphasizes both careful use during the encounter and targeted review after note generation. The approach is presented in Table 1, with brief descriptions below.

**Table 1:** The 3C approach to ambient scribe use

	Before the Encounter: Choose	During the Encounter: Capture	After Note Generation: Check
Clinician Task	Determine whether ambient scribe use is appropriate for this patient, encounter, and documentation task.	Optimize capture of clinically important information in usable form.	Perform targeted review of the generated draft.
Rationale	Some encounters, discussions, communication contexts, capture conditions, or documentation tasks may be poorly suited to ambient scribe use.	Information that is not clearly verbalized, captured, or attributed may be omitted, distorted, or underemphasized.	A fluent draft may contain omissions, unsupported or incorrect content, misattribution, incorrect findings, overstated certainty, misplaced emphasis, or unnecessary verbiage.
Practical Actions	Confirm disclosure and consent; assess discussion sensitivity, participants, communication context, capture conditions, and tool-note-specialty fit; select another documentation approach when fit is poor.	Verbalize key findings, reasoning, assessment, and plan; clarify ambiguity; identify speakers when needed; optimize microphone placement; pause capture when part of the encounter should remain outside the pipeline.	Compare the draft with the encounter; confirm key information is present, correctly attributed, and reflects the final version of corrected statements; remove unsupported or incorrect content; correct findings, certainty, and emphasis; trim unnecessary verbiage before signature.

Choose

Before the encounter, the clinician’s task is to determine whether ambient scribe use is appropriate for this patient, this encounter, and this documentation task. This includes confirming disclosure and consent, considering whether audio may be recorded or otherwise retained, assessing discussion sensitivity and participants, and judging whether the tool fits the clinical context, capture conditions, and expected note type [1-3,5,6,12,17,19,20,25,32,53,57,64]. Ambient scribe use may be well-suited to routine follow-up visits, medication reviews, stable chronic disease management, and straightforward history-based encounters in which key information is likely to be spoken clearly. Greater caution is warranted when encounters involve interpreters, multiple family members, cognitive impairment, emotional distress, limited English proficiency, dysarthria, poor acoustic conditions, heavy reliance on physical examination findings, or complex specialty reasoning that may not be fully verbalized. If the patient is uncomfortable with capture, the discussion is highly sensitive, or the encounter is otherwise a poor fit for the tool, another documentation method or temporary pause in capture may be more appropriate. Choosing another documentation method when fit is poor is not a rejection of ambient scribe technology; it is appropriate tool selection.

Capture

During the encounter, the clinician’s task is to optimize capture of clinically important information in usable form. Later stages operate on captured and processed input rather than on the encounter itself, so information that is not clearly verbalized, captured, or attributed may be omitted, distorted, or underemphasized. The clinician should verbalize key findings, reasoning, assessment, and plan; clarify ambiguity; identify speakers when needed; optimize microphone placement; and pause capture when part of the encounter should remain outside the pipeline. When information changes during the encounter, the correction should be verbalized explicitly, for example, by stating that an earlier medication dose, date, symptom report, or plan was incorrect and then clearly restating the final version [3,18,19,25,32,52,53].

Check

After note generation, the clinician’s task is targeted review of the draft. The draft is generated from an intermediate representation of the encounter, and fluency is not evidence of fidelity. A fluent draft may contain omissions, unsupported or incorrect statements, misattribution, incorrect findings, overstated certainty, misplaced emphasis, unnecessary verbiage, or an earlier statement that was superseded by a later correction. Review should focus on predictable artifact classes, including whether key information is present, correctly attributed, supported by the encounter, and consistent with the final version of the history, findings, assessment, and plan [11-13,15-17,54].

Summary of supporting evidence

To support interpretation of the evidence base, the conceptual domains, supporting sources, and role of each domain in this review are summarized in Table 2. Key findings from included ambient scribe evaluation studies are summarized in Table 3.

**Table 2:** Major conceptual domains and supporting sources

Conceptual Domain	Supporting Sources	Role in the Review
Mental models and clinician use of complex tools	[21-23]	Supports the argument that clinicians use complex tools more effectively when they have a functional mental model of how the tool operates.
Ambient scribe adoption, clinical implementation, and clinician experience	[1-10,20,24,25,30,57,64]	Supports discussion of increasing clinical use, potential effects on documentation burden and workflow, clinician experience, and implementation challenges.
Ambient scribe evaluation, quality, and safety	[1,2,4,10-17,24,32,54-57,64]	Supports discussion of observed note-quality issues, including omission, unsupported insertion or incorrect text, inaccurate or distorted content, speaker-attribution errors, excessive length, and editing burden.
Clinician editing behavior and documentation voice	[54,58]	Supports discussion of why clinicians modify generated drafts and how ambient scribes may affect documentation voice, clinical communication, and professional identity.
Privacy, consent, governance, and legal/ethical concerns	[3,17,19,20,59-63]	Supports discussion of meaningful consent, responsible implementation, cloud-based processing, data privacy, secondary use, governance, legal and ethical implications, and disclosure obligations.
Medicolegal liability and data retention	[17,19,60-62]	Supports discussion of clinician responsibility for signed documentation, malpractice exposure from inaccurate drafts, vendor data retention, and potential discovery of recordings, transcripts, drafts, metadata, or other artifacts.
Acoustic capture and digitization	[18,24,33-36]	Supports explanation of how microphones capture sound, convert acoustic energy into electrical signals, and transform continuous acoustic waveforms into sampled digital data.
Automatic speech recognition and transcript generation	[32,36-42,52,53]	Supports explanation of how digitized acoustic input is converted into written text through feature extraction, probabilistic modeling, and speech recognition methods, including known limitations and disparities.
Speech activity detection, speaker diarization, and attribution	[12,13,43,44]	Supports discussion of how systems identify speech segments, group speech by speaker, and may misattribute statements to the wrong participant.
Tokenization, language models, and draft note generation	[31,36,45-51]	Supports explanation of how transcript text is converted into numerical representations and transformed into draft documentation through language-model-based generation.
Pipeline-based understanding of artifact propagation	[11,12,16-18,24,32]	Supports the central claim that artifacts may arise at multiple stages of the pipeline and may persist, compound, or be reformulated downstream.
Practical clinician-use framework	[1-3,11-17,19,25,32,52-54,56,57,59-61,64]	Supports the practical framework for clinician judgment before use, deliberate communication and capture practices during the encounter, and targeted review of predictable artifacts after note generation.

**Table 3:** Findings from referenced ambient scribe evaluation studies EHR: Electronic health record; SOAP: Subjective, objective, assessment, and plan; ADS: Ambient digital scribe; PDQI: Physician Documentation Quality Instrument

Study	Sample/Setting	Main Quantitative Findings	Context for Interpretation
Duggan et al. [5]	46 clinicians; outpatient academic health system improvement study	Ambient scribe use associated with 20.4% less time in notes per appointment, 9.3% greater same-day appointment closure, 30.0% less after-hours work time per workday.	Single health system; pre-post design; outpatient setting; clinician experience included both favorable and mixed responses.
North et al. [6]	332 primary care physicians; Epic Signal documentation metrics before and after implementation	Use increased from 15% to 50% of physicians over 8 weeks; mean time in notes decreased from 5.11 to 4.16 minutes per note; note length increased by 238 characters.	Primary care only; EHR-derived metrics; short observation window.
Ma et al. [7]	45 physicians; 8 ambulatory disciplines; 3-month improvement pilot	Tool used in 9,629 of 17,428 encounters; median time per note decreased by 0.57 minutes; median daily documentation time, after-hours time, and total EHR time decreased by 6.89, 5.17, and 19.95 minutes/day, respectively.	Single academic medical center; pilot implementation; individual-level heterogeneity.
Olson et al. [8]	263 ambulatory clinicians across 6 health systems; pre-post survey after 30 days	Burnout decreased from 51.9% to 38.8%; significant improvements in cognitive task load, time spent documenting after hours, focused attention on patients, and ability to add urgent patients.	Quality improvement design; voluntary participation; short follow-up; survey-based.
You et al. [9]	1430 clinicians across Mass General Brigham and Emory Healthcare; survey study	Burnout decreased from 52.6% to 30.7%	Survey-based; response-rate and selection-bias concerns; clinician-reported documentation burden and benefit.
Rotenstein et al. [10]	8,581 clinicians; 1,809 adopters vs 6,772 non-adopters; 5 academic medical centers	Ambient scribe adoption associated with 13.4 fewer minutes of EHR time and 16 fewer minutes of documentation time per 8 scheduled patient hours, and 0.49 additional weekly visits; no significant change in after-hours EHR time.	Observational study; voluntary adoption; benefits varied by clinician group and intensity of use.
Anderson et al. [11]	5 ambient platforms; 14 simulated ambulatory encounters	Mean 26.3% of key clinical elements omitted or captured erroneously; omissions accounted for 76.3% of errors; 19.5% of transcript errors transmitted to notes.	Simulated ambulatory encounters; limited specialty range; platform-level variability.
Lukac et al. [13]	238 physicians randomized to two AI scribe tools or control	Modest reduction in time-in-note for one tool; burnout, work exhaustion, and task load showed no clear improvement with use of either scribe.	Randomized trial; vendor-specific effects; short implementation window.
Ha et al. [14]	6 ambient scribe products evaluated using 4 standardized primary care encounters	No products were consistently error-free; common errors included deletion, omission, and SOAP structure errors.	Controlled competitive analysis; simulated encounters; vendor identities masked; not a real-world clinical implementation.
Brunner et al. [15]	Simulated patient encounters across 4 specialties and 2 products; Modified PDQI-9 note-quality assessment	Mean score of Product A:38.3/50; mean score of Product B: 33.1/50.	Simulated environment; small sample; modified scale led to elevated scores; cross-specialty exploratory design.
Biro et al. [16]	44 draft notes; 2 commercial ADS products; 22 simulated encounters per product	70% of draft notes contained at least 1 error; mean 2.9 errors per note.	Simulated encounters; 2 products; ambulatory focus; designed to identify error types and safety concerns.
Leiserowitz et al. [20]	1,893 patients, preimplementation survey, academic medical center	48% favorable anticipated responses to ambient scribes, 33% neutral, 19% unfavorable; open-ended comments emphasized improved human connection but raised concerns about accuracy and privacy.	Patient attitudes before implementation; 20% response rate; respondents not fully representative of clinic population.
Palm et al. [55]	97 patient visits across 5 specialties; ambient scribe generated notes compared with specialist-drafted notes using PDQI-9	Ambient notes more thorough and better organized, but less succinct and more prone to hallucination.	Blinded note-quality study; focused on note quality rather than workflow, safety events, or implementation outcomes.
van Buchem et al. [56]	Single center; single product; usability and note-quality study using PDQI-9	Median time saving 16 seconds per visit; ambient scribe notes scored lower on PDQI-9 than manual notes, with higher word count and lower lexical diversity.	Simulated encounters; single center and product; students rather than practicing clinicians; not a safety or accuracy study.
Shah et al. [57]	Physician survey; multiple sites and specialties	Net positive scores highest for cognitive and time demand, work-life integration, workload, future use, and patient engagement; net negative scores for accessibility, note construction, completeness, accuracy, formatting, length, style, and editing requirements.	Self-report; cross-sectional; selection bias possible; focused on adoption and usability themes.

## Conclusions

Ambient scribes can produce fluent and clinically useful draft documentation. Their output is best understood as a machine-mediated representation of the clinical encounter produced through successive computational transformations. This perspective clarifies both the usefulness and the limitations of these tools. The same pipeline that allows captured sound to become a structured draft note also helps explain why note accuracy, workflow fit, privacy, consent, and clinician responsibility must be considered together when these tools are used in clinical care.

The evidence base presented in this review should be interpreted with restraint. The literature on component processes such as acoustic capture, digitization, speech recognition, speaker attribution, and language-model-based text generation is much more mature than the literature evaluating ambient scribes as end-to-end clinical products. Existing clinical studies suggest potential benefit, but the evidence remains uneven across products, specialties, settings, patient populations, and evaluation methods. It is therefore possible to describe the general pipeline with greater confidence than it is possible, at present, to make precise claims about the performance, safety, or use of specific commercial products across diverse clinical contexts.

As the technology matures, broader evaluation should clarify which tools work best for which clinicians, patients, specialties, and encounter types. Statutory and regulatory guidelines governing consent, data retention, and liability are likely to evolve. Until then, a mechanistic understanding of the pipeline offers a stable basis for clinician judgment. The pragmatic response is neither uncritical acceptance of fluent output nor reflexive rejection of the technology. It is disciplined use: choosing when ambient scribe use is appropriate, capturing clinically important information in usable form, and checking the generated draft for predictable artifacts before signature.
